# An ecological approach to binocular vision

**DOI:** 10.1177/20416695221103895

**Published:** 2022-06-07

**Authors:** Barbara Gillam

**Affiliations:** The University of New South Wales, Sydney, Australia

**Keywords:** 3D perception, binocular vision, stereopsis, scene perception

## Abstract

An ecological approach to binocular vision was already demonstrated in Wheatstone's initial stereograms and was explicitly called for by J. J. Gibson, but detailed analysis and experimentation supporting this approach has been more recent. This paper discusses several aspects of this more recent research on environmentally occurring spatial layouts that can influence binocular vision. These include gradients of depth and regions that can be seen by only one eye. The resolution of local stereoscopic ambiguity by more global factors is also discussed.

## Introduction

Stereopsis has tended to be studied within a largely physiological tradition based on “binocular disparity” defined as the difference in the location of retinal points in the two eyes produced by two points in space located at different depths. In the following paper, I shall describe several ways in which elements of a depth-varying scene can present to the two eyes and demonstrate that the visual responses to binocular input go well beyond processing conventional binocular disparity. In other words, I shall attempt a more ecological account of the information used in binocular viewing.

**Figure 1.**
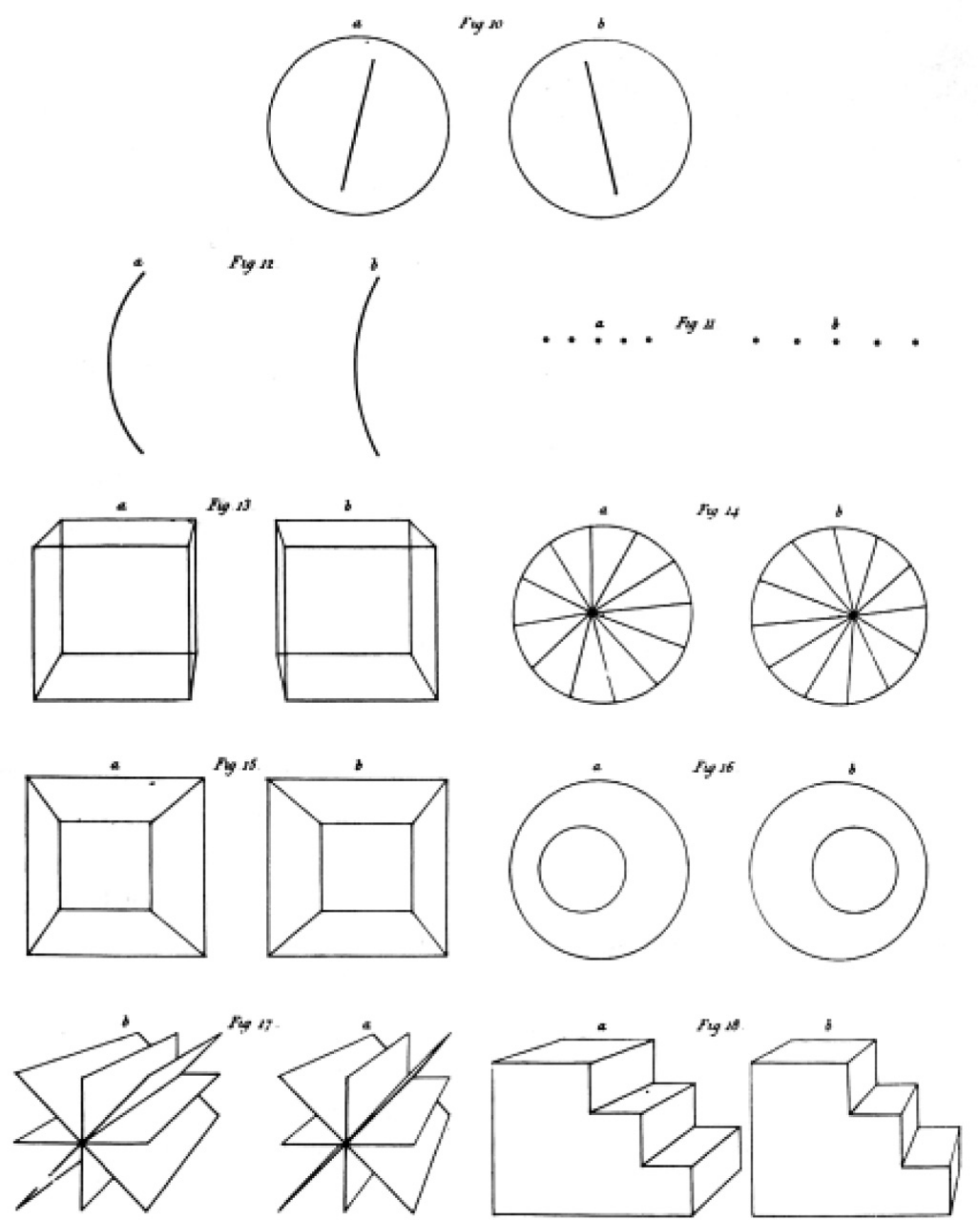
Wheatstone stereograms. Adapted from Wheatstone (1838).

Figure 1 shows some Wheatstone stereograms (Wheatstone, 1838), which illustrate many ways in which the images from objects or arrays with components in depth can differ in the two eyes. For many years, these complexities were neglected in the study of binocular vision in favor of a highly physiological approach based on simple line stimuli and the “horopter” or the mapping of corresponding points in the two eyes.

The best known of the differences in binocular images is width disparity. However, more complex disparities in orientation, curvature and spatial frequency can also be seen in Figure 1. These may have direct visual effects rather than being reduced to width disparities (although such reduction is theoretically possible).

## Binocular Stereopsis in the Work of J. J. Gibson

J. J. Gibson did little if any research on binocular stereopsis. His only extended discussion of it is in *The Perception of the Visual World* (Gibson, 1950, pp. 100–108). It appears that the main goal of that discussion is to assimilate binocular stereopsis into his overall theory that visual space perception is based on the perception of the extended 3D surfaces of the environment. In that theory, the perception of a surface receding in depth arises principally from three gradients in the optical projection of the surface to the eyes: (1) a gradient of texture size (or density), (2) a gradient of motion parallax, and (3) a gradient of binocular disparity. Gibson saw these gradients as closely related to each other in that each was an expression of the broader principle of perspective diminution with distance. Gibson's emphasis, in his own research, on the gradients of texture and of motion may have arisen in part from a wish to counteract the then prevailing view that saw stereopsis (along with accommodation and convergence of the eyes) as being primary to space perception, with everything else relegated to the role of secondary cues (Gibson, 1950, p. 72). In addition, Gibson's early research on the visual space perception of airplane pilots was concerned with large distances, at which, he believed, binocular stereopsis was of little use (Gibson, 1947, pp. 181–182).

For Gibson, an important similarity between the gradient of motion parallax and the gradient of binocular disparity is that both gradients are maximal at their closest point to the observer and decrease gradually toward a minimum at the farthest location on the surface. Because an observer's eyes are mobile; however, the minimal motion on the retina will be at whatever distance the eye is fixating and tracking; likewise, the minimal retinal disparity will be at whatever distance the two eyes are fixating and converging on. Consequently, for Gibson, neither gradient can be defined simply by retinal motion or retinal disparity. Instead, Gibson initially referred to *relative* retinal motion and *relative* retinal disparity, which basically subtracted out the rotations of the eyes (Gibson, 1947, p. 225 and p. 194, respectively); this approach was a forerunner of his concept of the optic array, in which the structure of the light reaching a station point is defined independently from the eye that might be located there. These relations – between motion and stereopsis, and between retinal and optic array gradients – are a source of confusion in the contemporary literature, as I have discussed in more detail elsewhere (Gillam, 2007).

Another complexity that commonly exists in natural, crowded environments is that an observer's view of a surface is often partially occluded by another, closer surface. An important focus of Gibson's later work was both the information for occlusion that is carried by motion transformations and the ability of observers to make use of this information (Gibson 1966, 1979; Gibson et al., 1969). In particular, as an observer's point of observation moves, a farther surface can be progressively occluded or revealed. An analogous effect of occlusion occurs with binocular stereopsis, although it involves the static spatial separation of the two eyes rather than one eye's motion over time. Because of this spatial separation, more of a partially occluded surface may be visible to one eye than to the other; this portion of the surface is only visible monocularly and thus creates no traditional binocular disparity. Nevertheless, such monocular regions carry information about complex spatial layouts involving occlusion. My colleagues and I, as well as others, have experimentally explored a number of such complex situations, in which we have found that observers can correctly perceive occlusion, with quantitatively appropriate depth. Gibson, as far as I know, was unaware of these possibilities, but these discoveries are very much in line with an ecological approach. In what follows, I briefly describe three examples of our work (a more detailed discussion is available in Gillam, 2011).

## Stereoscopic Ambiguity – Slant or Occlusion?

**Figure 2.**
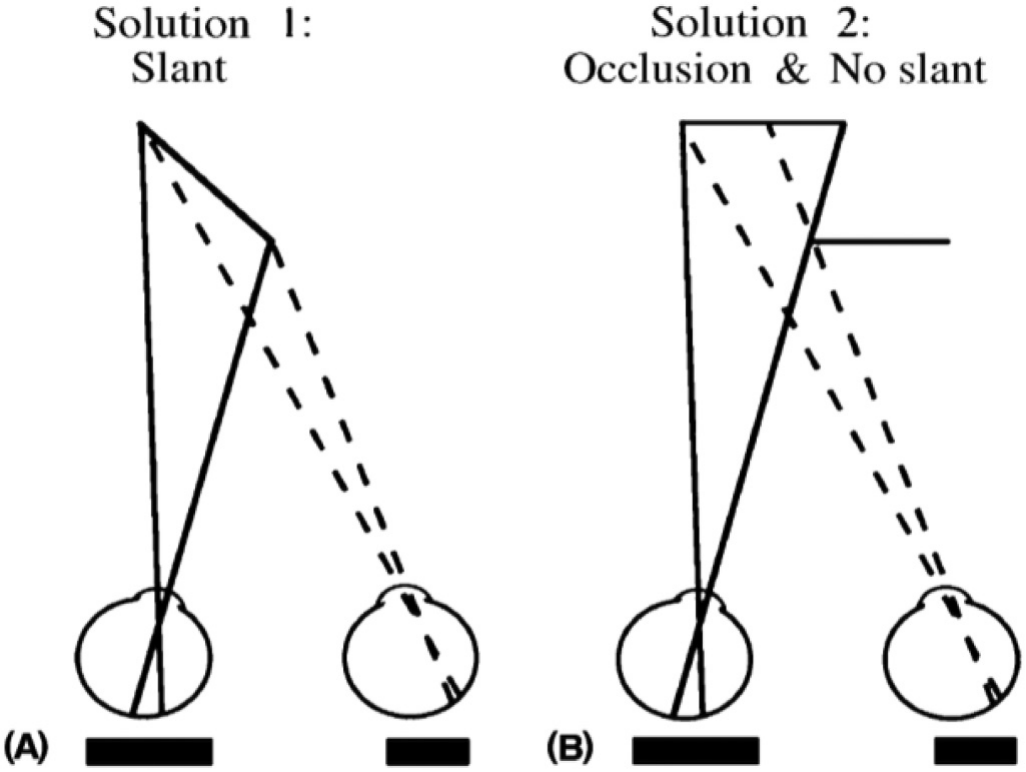
The same horizontal disparity can be produced by either a slant (on the left) or by the partial occlusion of one eye's image (on the right). Adapted from Gillam and Grove (2004).

The least discussed disparity in 3D images is produced by differential occlusion of a contour by a nearer surface. Wheatstone's example of this is shown in the bottom left pair of Figure 1. Figure 2 shows how the same horizontal disparity can be produced either by surface slant or by partial occlusion by a foreground surface.

**Figure 3.**
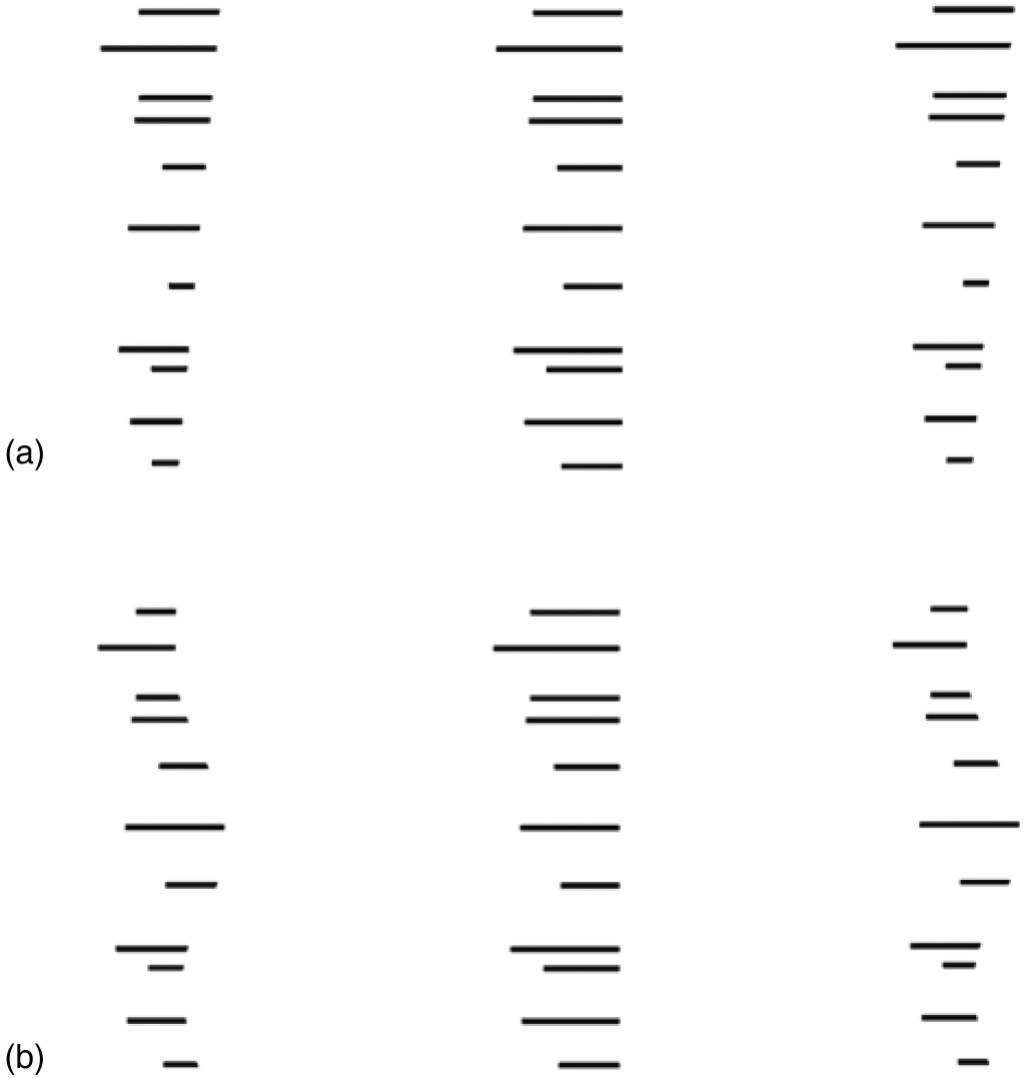
(a) Cross fusion of the left and center pair produces a subjective contour inclined in depth. Fusion of the center and right pair produces no subjective contour in depth; instead, the lines appear at various slants. (b) Cross fusion of the left and center pair produces a subjective contour curved in depth. Fusion of the center and right pair produces no subjective contour in depth; instead, the lines appear at various slants. Adapted from Gillam and Grove (2004).

Although the disparity of one line does not distinguish between an origin in slant or in occlusion, Figure 3 shows that the disparity pattern among sets of multiple lines of varying width can do so. In Figure 3, the left pair of truncated lines (in both a and b) is formed by a graded subtraction of part of each line, which is consistent with the presence of an occluding surface on the right side (seen here only as a subjective contour), whereas the differences in the widths of the right pair in each case are proportional to the line widths (consistent with magnification of one eye's view). The latter case can only be accounted for by individual slants of the lines, which is what is seen (Gillam & Grove, 2004).

## The Ecological Significance of Monocular Regions in Binocular Vision

### Binocular Responses to Monocular Gaps

**Figure 4.**
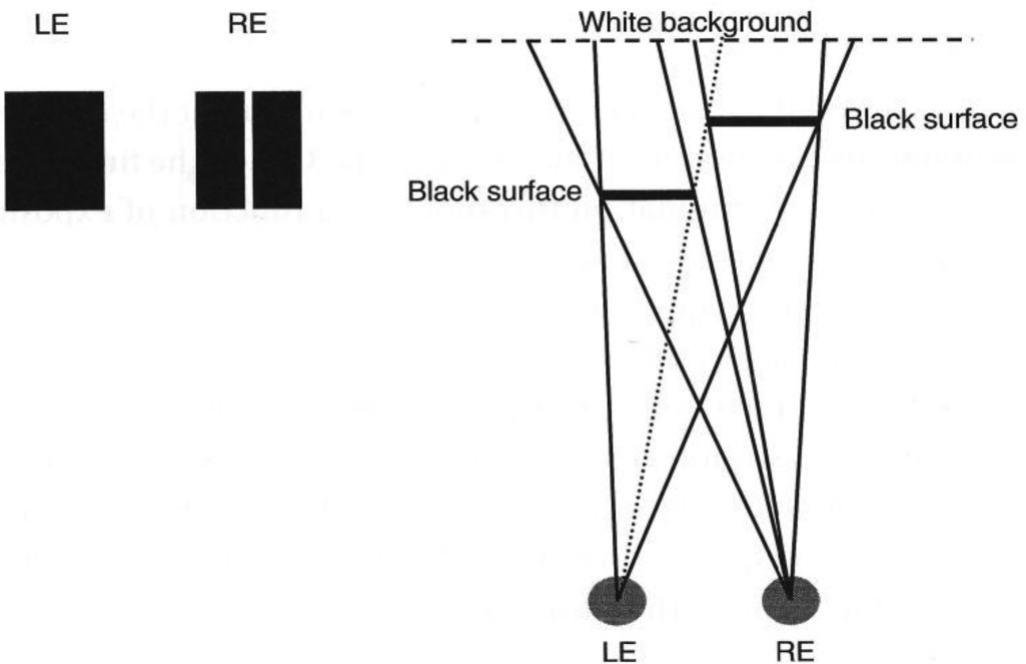
Stereogram (left) and bird’s eye view (right) of monocular gap stereopsis. Adapted from Gillam (2011).

Figure 4 on the left shows a stereogram of the layout that is illustrated in bird's eye view on the right. In this case, only the right eye can see part of the white background between the two black surfaces. The left eye cannot.

**Figure 5.**
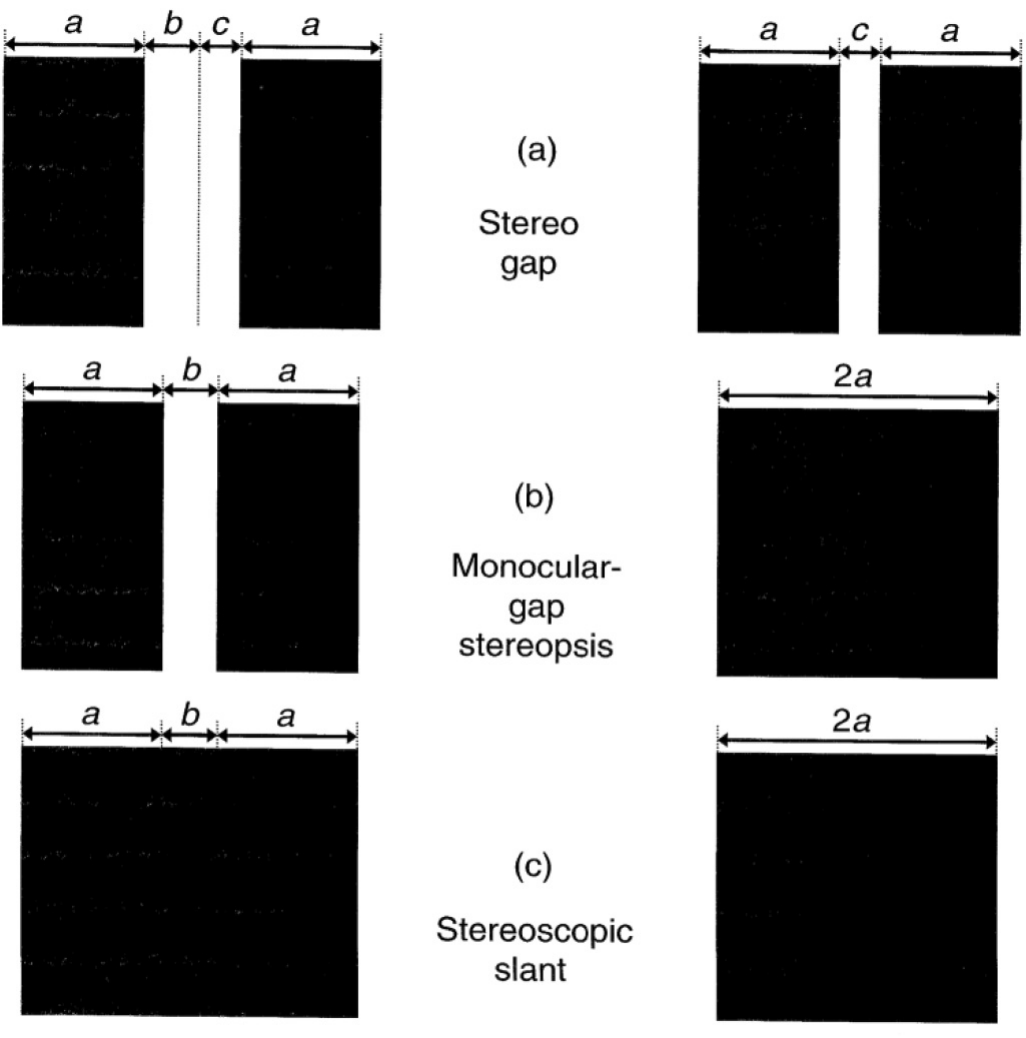
Three stereoscopic conditions. Adapted from Gillam (2011).

**Figure 6.**
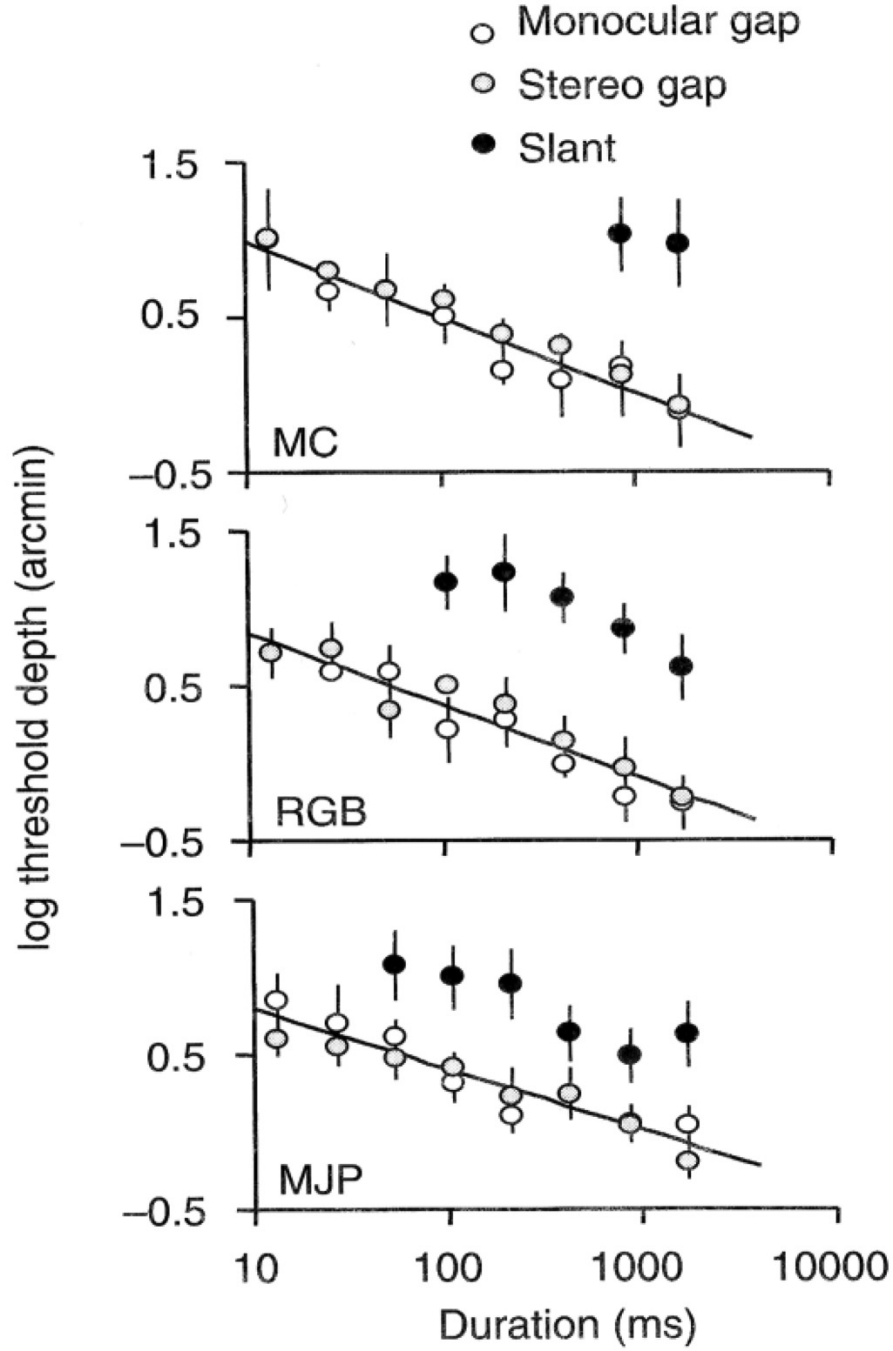
Comparing conditions with monocular gaps, stereoscopic gaps and slant on binocular depth detection thresholds as a function of stimulus duration. Adapted from Gillam (2011).

Figure 5 shows three figures used in an experiment (Pianta & Gillam, 2003) comparing the stereoscopic thresholds for detecting the depth seen at monocular and binocular gaps in binocular stimuli. Figure 5a is a regular stereogram with a disparate gap consistent with a depth difference between two surfaces. Figure 5b is a stereogram representing a surface placement such that only one eye can see the gap (monocular gap condition), whereas Figure 5c has only edge disparity, consistent with slant. The depth threshold data for each of these cases are shown in Figure 6. Fusion of each pair in Figure 5 shows that the monocular gap condition behaves like the stereo gap condition, suggesting that the solid figure is implicitly viewed as two adjoined figures, each fused with the separate images in the other eye. The data in Figure 6 support this view in that the functions for monocular gap and stereo gap overlap and are very different from the function for slant.

### Phantom Surfaces Produced by Uniocular Occlusion

**Figure 7.**
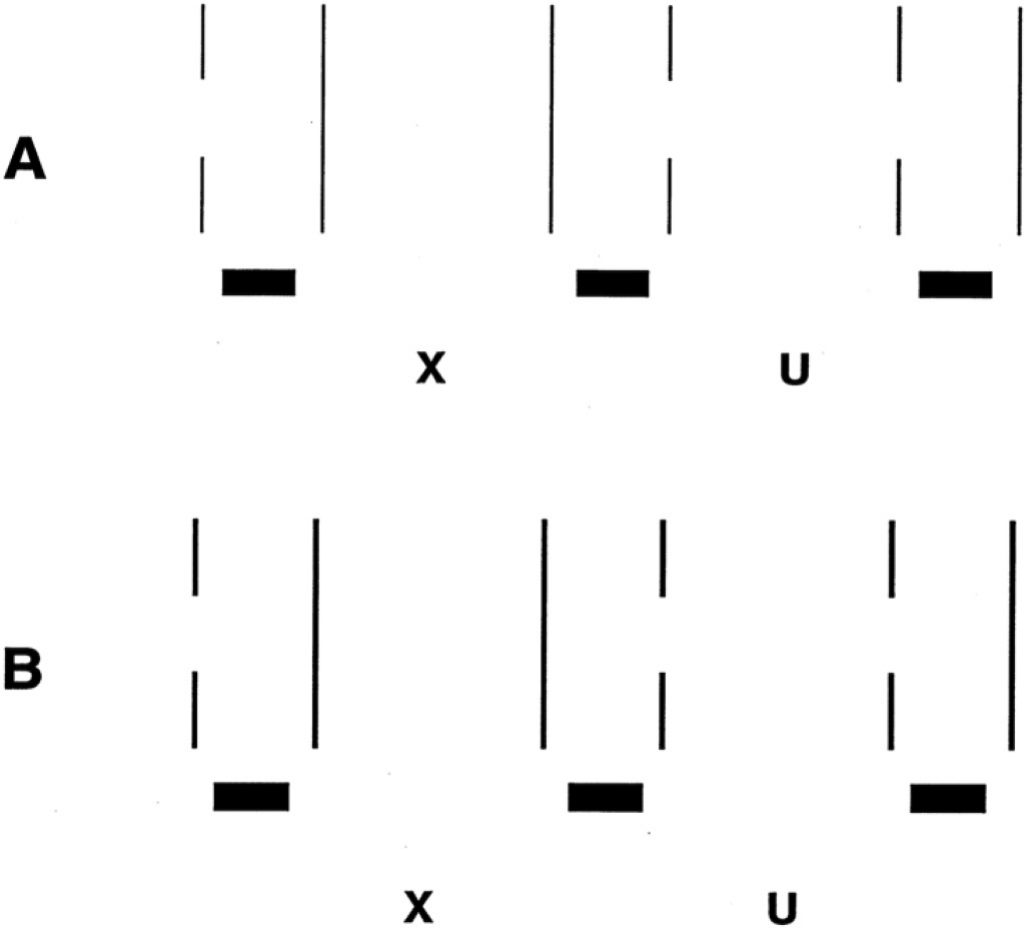
Stereogram seen as a “phantom” rectangle in front of two vertical lines. Vertical lines in (B) are twice as thick as vertical lines in (A). Adapted from Gillam and Nakayama (1999).

**Figure 8.**
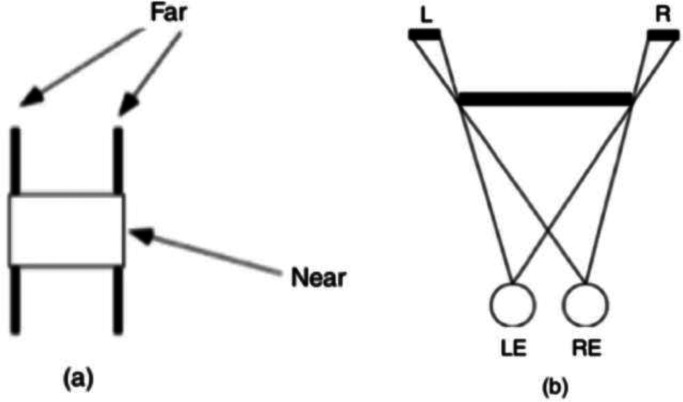
(a) An illustration of what is seen in Figure 7. (b) A bird’s eye view of the layout producing phantom stereopsis. Adapted from Gillam (2011).

Another striking ecological effect of monocular regions is produced by the stereogram shown in Figure 7. There is no positional disparity between the vertical lines in the left eye view and right eye view, but the right eye view has a gap in the left line and the left eye view has a gap in the right line. As is shown in the bird's eye view diagramed in Figure 8b, this pattern of binocular differences could result from the partial occlusion of each line by a surface floating in front of them. Figure 8a shows the cyclopean view of such a spatial layout. This is just what is seen; a “phantom surface” is perceived in front of, and partially occluding, the two vertical lines. Moreover, the depth seen in the stereogram is quantitatively related to the width of the occluded regions (i.e., the thickness of the lines) (Gillam & Nakayama, 1999).

## Conclusions

The examples discussed here show that binocular vision includes processes that respond to monocular regions in binocular displays in an ecologically appropriate way. In these examples, the presence of monocular regions did not result in suppression or rivalry but in a perceived spatial layout in which the gap between two areas exists but would indeed not be visible in one eye's view. Although not known to Gibson, these results offer striking support of the ecological approach to visual perception.

## References

[bibr1-20416695221103895] Gibson J. J. (1947). *Motion picture testing and research. Aviation Psychology Research Reports, No. 7, U.S. Government Printing Office* .

[bibr2-20416695221103895] Gibson J. J. (1950). The perception of the visual world. Houghton Mifflin.

[bibr3-20416695221103895] Gibson J. J. (1966). The senses considered as perceptual systems. Houghton Mifflin.

[bibr4-20416695221103895] Gibson J. J. Kaplan G. A. Reynolds H. N. Jr. Wheeler K. (1969). The change from visible to invisible: A study of optical transitions. Perception & Psychophysics, 5, 113–116. 10.3758/BF03210533

[bibr5-20416695221103895] Gibson J. J. (1979). The ecological approach to visual perception. Houghton Mifflin.

[bibr6-20416695221103895] Gillam B. (2007). Stereopsis and motion parallax. Perception, 36, 953–954. 10.1068/p3607ed 17844961

[bibr7-20416695221103895] Gillam B. (2011). The influence of monocular regions on the binocular perception of spatial layout. In Harris L. R. Jenkin M. R. M. (Eds.), Vision in 3D environments (pp. 1–14). Cambridge University Press.

[bibr8-20416695221103895] Gillam B. Grove P. M. (2004). Slant or occlusion: Global factors resolve stereoscopic ambiguity in sets of horizontal lines. Vision Research, 44, 2359–2366. 10.1016/j.visres.2004.05.002 15246752

[bibr9-20416695221103895] Gillam B. Nakayama K. (1999). Quantitative depth for a phantom surface can be based on cyclopean occlusion cues alone. Vision Research, 39, 109–112. 10.1016/S0042-6989(98)00052-2 10211399

[bibr10-20416695221103895] Pianta M. J. Gillam B. J. (2003). Monocular gap stereopsis: manipulation of the outer edge disparity and the shape of the gap. Vision Research, 43, 1937–1950. 10.1016/S0042-6989(03)00252-9 12831756

[bibr11-20416695221103895] Wheatstone C. (1838). Contributions to the physiology of vision: I. On some remarkable and hitherto unobserved phenomena of binocular vision. Philosophical Transactions of the Royal Society, 128, 371–394. 10.1098/rstl.1838.0019

